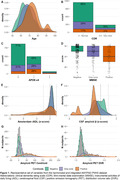# From AMYPAD to Euro‐PAD: creating a multi‐cohort preclinical database to model onset of Alzheimer’s disease

**DOI:** 10.1002/alz.091384

**Published:** 2025-01-09

**Authors:** David Vállez García, Lyduine E. Collij, Willemijn J. Jansen, Stephanie J. B. Vos, Julie Elisabeth Oomens, Natalia Vilor‐Tejedor, Laura Stankeviciute, Marta Mila‐Aloma, Anna E Leeuwis, Gonzalo Sánchez‐Benavides, Mahnaz Shekari, Fiona Heeman, Luigi Lorenzini, Leonard Pieperhoff, Bert Overduin, Isadora Lopes Alves, Juan Domingo Gispert, Frank Jessen, Pieter Jelle Visser, Anouk den Braber, Craig W Ritchie, Mercè Boada, Marta Marquié, Rik Vandenberghe, Emma S. Luckett, Michael Schöll, Giovanni B. Frisoni, Bernard J Hanseeuw, Lisa Quenon, Andrew W. Stephens, Lisa Ford, Mark E Schmidt, Cindy Birck, Anja Mett, Rossella Gismondi, Richard Manber, Sylke Grootoonk, Robin Wolz, Gill Farrar, Frederik Barkhof, the AMYPAD Consortium

**Affiliations:** ^1^ Amsterdam UMC, Amsterdam Netherlands; ^2^ Lund University, Lund Sweden; ^3^ Maastricht University, Maastricht Netherlands; ^4^ Alzheimer Center Limburg, School for Mental Health and Neuroscience, Maastricht University, Maastricht Netherlands; ^5^ Barcelonaβeta Brain Research Center (BBRC), Barcelona Spain; ^6^ Barcelonaβeta Brain Research Center (BBRC), Pasqual Maragall Foundation, Barcelona Spain; ^7^ University of Gothenburg, Gothenburg Sweden; ^8^ Aridhia Informatics Ltd, Glasgow UK; ^9^ Brain Research Center, Amsterdam Netherlands; ^10^ Barcelonaβeta Brain Research Center (BBRC), Pasqual Maragall Foundation, Barcelona Spain; ^11^ German Center for Neurodegenerative Diseases (DZNE), Bonn Germany; ^12^ Scottish Brain Sciences, Edinburgh UK; ^13^ Ace Alzheimer Center, Barcelona Spain; ^14^ Ace Alzheimer Center Barcelona, Barcelona Spain; ^15^ University Hospitals Leuven, Leuven Belgium; ^16^ Karolinska Institutet, Stockholm Sweden; ^17^ University of Geneva, Geneva Switzerland; ^18^ Institute of Neuroscience ‐ UCLouvain, Brussels Belgium; ^19^ Institute of Neuroscience, UCLouvain, Brussels Belgium; ^20^ Life Molecular Imaging GmbH, Berlin Germany; ^21^ Janssen Pharmaceutica, Titusville, NJ USA; ^22^ Janssen Pharmaceutica, Beerse Belgium; ^23^ Alzheimer Europe, Luxembourg Luxembourg; ^24^ GE Healthcare, Amersham UK; ^25^ IXICO, London UK; ^26^ IXICO, London, Greater London UK; ^27^ GE HealthCare, Amersham UK; ^28^ University College London, London UK

## Abstract

**Background:**

The emergence of disease‐modifying drug therapies is expected to revolutionize the field of Alzheimer's disease (AD). Recent results from anti‐amyloid clinical trials highlight the importance of early identification and accurate risk‐stratification of individuals in early stages of the disease. In this context, the Amyloid Imaging to Prevent Alzheimer’s Disease (AMYPAD) Prognostic and Natural History Study (PNHS) was established, leveraging existing cohorts to alleviate the burden of recruiting de novo participants. Here, we describe the harmonization and integration efforts of brain imaging, clinical, cognitive, and fluid biomarker data.

Access to the data is available through the Alzheimer’s Disease Data Initiative, with additional details provided at https://amypad.eu/data/

**Method:**

The AMYPAD PNHS integrates prospective and historical data from 32 European sites across 10 countries. These sites contribute data from 10 Parent Cohorts (PC), predominantly comprising non‐demented at‐risk subjects, including EPAD LCS, EMIF‐AD (60++ and 90+), ALFA+, FACEHBI, FPACK, UCL‐2010‐412, Microbiota, DELCODE, and the AMYPAD Diagnostic and Patient Management Study.

A meticulous data curation process was implemented, harmonizing metrics and questionnaires through strategies such as recoding into categories, Percentage of Maximum Possible Scores, and z‐scores. Expert reviewers at each site conducted PET visual reads. Centralized quantification of static PET images, employing site‐specific Gaussian smoothing, yielded harmonized Centiloid values. Parametric modelling of dynamic PET scans was also performed providing metrics such as the distribution volume ratio.

**Result:**

The initial data set includes 3366 participants (55% females, 67±8 years), with 2629 having at least one follow‐up visit (2.6±1.9 years). Of those, 1618 underwent baseline amyloid PET, 888 with follow‐up. The dataset incorporates clinical outcomes, biomarkers, risk factors, and other relevant variables (Figure 1). Distribution of participants based on amyloid PET status at baseline yielded 60% negative (<12CL), 24% grey‐zone (12‐50CL), and 16% positive (>50CL) cases.

**Conclusion:**

The AMYPAD PNHS represents the largest European longitudinal dataset phenotyping individuals at risk of AD‐related progression. The consortium is currently evolving into its new phase, namely the Euro‐PAD collaborative framework, and the dataset will be expanded in terms of variables (e.g., currently integrating GWAS and advanced MRI data) and number of cohorts. Interested to join, please contact us at https://amypad.eu/